# Circulating betatrophin is associated with insulin resistance in humans: cross-sectional and interventional studies *in vivo* and *in vitro*

**DOI:** 10.18632/oncotarget.21852

**Published:** 2017-10-16

**Authors:** Han Wang, Lin Du, Tong Wu, Gangyi Yang, Wenjing Hu, Hansheng Wang, Mengliu Yang, Dongfang Liu, Harvest F. Gu, Zhiming Zhu, Hongting Zheng, Ling Li

**Affiliations:** ^1^ The Key Laboratory of Laboratory Medical Diagnostics in The Ministry of Education and Department of Clinical Biochemistry, College of Laboratory Medicine, Chongqing Medical University, Chongqing, China; ^2^ Department of Endocrinology, The Second Affiliated Hospital, Chongqing Medical University, Chongqing, China; ^3^ Department of Clinical Science, Intervention and Technology, Karolinska Institutet, Karolinska University Hospital, Huddinge, Stockholm, Sweden; ^4^ Center for Molecular Medicine, Karolinska Institute, Karolinska University Hospital, Solna, Stockholm, Sweden; ^5^ Department of Hypertension and Endocrinology, Daping Hospital, Third Military Medical University, Chongqing Institute of Hypertension, Chongqing, China; ^6^ Department of Endocrinology, Xinqiao Hospital, Third Military Medical University, Chongqing, China

**Keywords:** betatrophin, insulin resistance, metabolic disorders, interventional study, cell cross-talk, Gerotarget

## Abstract

Betatrophin has a closely relationship with metabolism. However, its effect on metabolism disorder remains unclear. This study was comprised of a series of cross-sectional and interventional studies *in vivo* and *vitro*. PCOS women with IR and healthy women were recruited from the general population and outpatients. Plasma betatrophin levels were measured with ELISA. Insulin sensitivity was assessed with EHC. Gene expressions at mRNA and protein levels were determined with RT-PCR and Western blotting. Influences of insulin, metformin, rosiglitazone and over- or knockdown-expression of betatrophin were analyzed *ex vivo*. Our results indicated that IR women had higher betatrophin levels compared with the controls. Circulating betatrophin was positively correlated with BMI, WHR, Fat%, triglyceride, total cholesterol, LDL-C, AUC_glucose_ and AUC_insulin_, luteinizing Hormone, FAI and HOMA-IR but negatively with M-value. Metformin treatment in PCOS women with IR led to a reduction of betatrophin levels. Insulin stimulation in hepatocytes increased betatrophin expression. Metformin or rosiglitazone led to a reduction of betatrophin expression in insulin-stimulated hepatocytes. In hepatocytes/macrophages co-culture systems, betatrophin expression was significantly increased, whereas this increase was eliminated by rosiglitazone. In hepatocytes, overexpression and knockdown of betatrophin decreased or increased insulin-stimulated insulin receptor, protein kinase B and insulin receptor substrate-1 phosphorylation respectively. Serum from metformin-treated women with IR decreased betatrophin expression and reinforced insulin signals. Thus, the present study provides the *in vivo* and *in vitro* evidence, suggesting that there is a cell cross-talking between hepatocytes with macrophages for the regulating betatrophin and it may be a useful marker for IR and metabolic disorders.

## INTRODUCTION

Betatrophin, also known as angiopoietin-like protein (ANGPTL) 8, is highly conserved in all mammalian species [1]. This protein is predominantly expressed in liver and fat tissues, and its hepatic expression is found to be increased in rodent models of insulin resistance (IR) [2-4]. Betatrophin was primordially reported as a regulator of β cell proliferation [4]. Moreover, circulating betatrophin levels were increased in the subjects with type 1 diabetes and insulin-deficient mice [4, 5]. In mouse models of type 2 diabetes mellitus (T2DM), however, expression of betatrophin in liver was upregulated. Specific depletion of beta-cells did not cause betatrophin upregulation, suggesting that betatrophin levels might be regulated by IR and not insulin deficiency [5]. Several studies demonstrated that circulating betatrophin levels were increased in the subjects with T2DM and obesity [6-8], while Gómez-Ambrosi *et al.* reported that betatrophin levels were decreased in T2DM and obesity [9]. Furthermore, the reports concerning the correlation between betatrophin and insulin and/or atherogenic lipid profiler were also inconsistent [7-10]. Therefore, it is necessary to further investigate the effects of betatrophin on metabolic disorders.

Polycystic ovary syndrome (PCOS) is characterized by heterogeneity in phenotypic manifestations mainly related to reproductive and hormone aberrations and metabolic disturbances [11-13]. IR and metabolic disorders are the most significant and highly prevalent parameter among PCOS women [14-16]. Hyperinsulinemia is more prevalent in lean and obese PCOS women than in age-matched healthy women [17]. However, unlike T2DM, PCOS is a condition characterized by IR and metabolic disturbances in lean and obese women and may represent an adequate model to study the relationship of betatrophin and IR independent of the effect of severe hyperglycemia observed in T2DM.

In the present study, we first measured circulating betatrophin concentrations in IR and healthy women. Secondly, we evaluated the effects of fasting (a low-insulin and low-glucose state), an oral glucose tolerance test (OGTT, a highglucose state) and euglycemic–hyperinsulinemic clamp (EHC, a euglycemic–hyperinsulinemic state) on betatrophin levels. Thirdly, we examined whether circulating betatrophin levels are changed by metformin administration. In *vitro* study, we assessed the effects of insulin, metformin or rosiglitazone on betatrophin expression and investigated the role of overexpression or knockdown of betatrophin on insulin signal pathway. Finally, we observed the effects of serum from metformin-treated IR subjects on betatrophin expression in hepatocytes.

## RESULTS

### Circulating betatrophin levels and their association with anthropometric and biochemical parameters in study population

**Figure 1:**
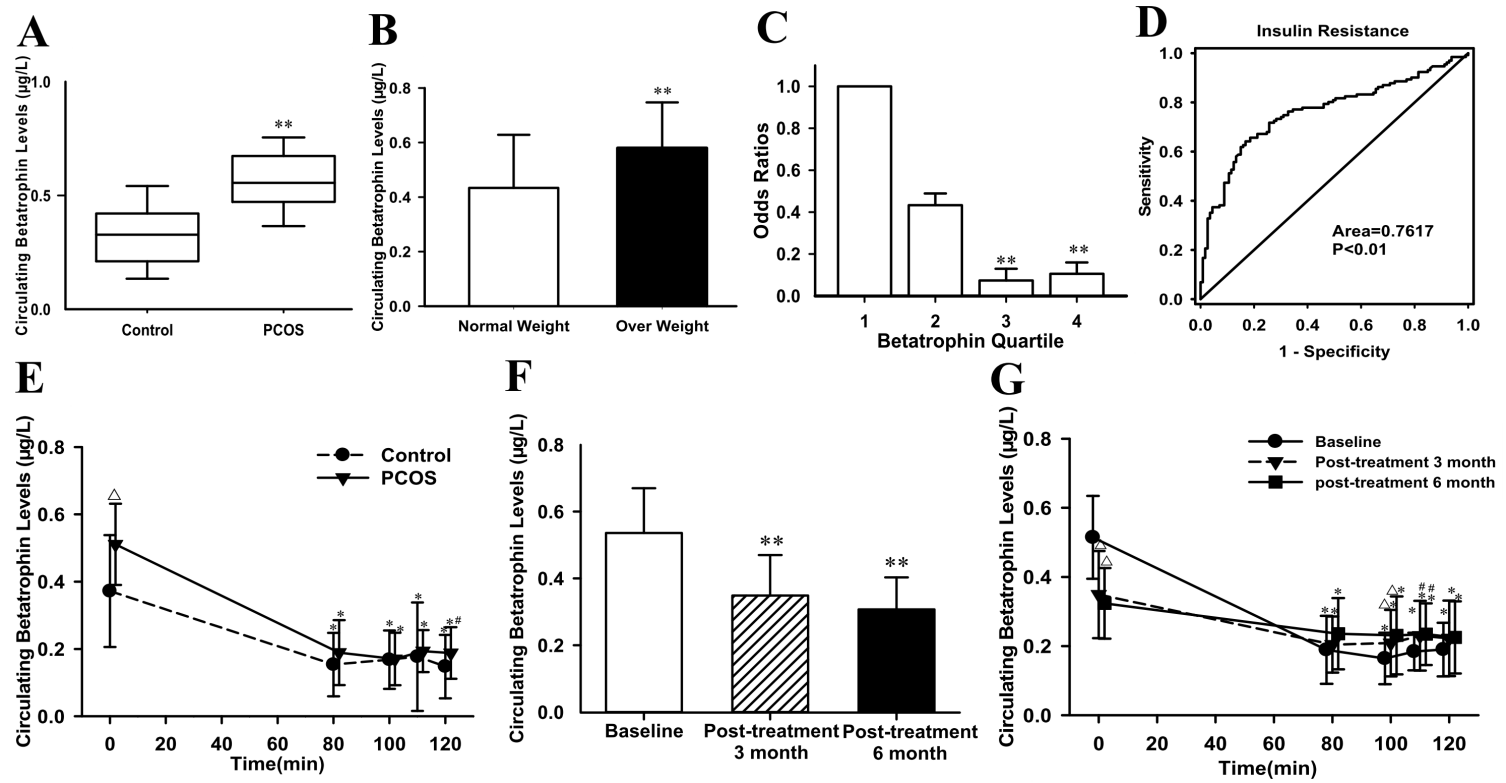
Circulating betatrophin levels and ROC curve analysis in the study cohort (**A**) Circulating betatrophin levels in normal and PCOS women with IR (*vs.* controls: ** *P* < 0.01). (**B**) Circulating betatrophin levels according to BMI (normal-weight: BMI < 25kg/m^2^ and overweight/obese: BMI >25 kg/m^2^, *vs.* normal weight: **P < 0.01). (**C**) Prevalence of elevated PCOS in different quartiles of betatrophin: quartile 1, <0.33μg/L; quartile 2, 0.33-0.49μg/L; Quartile 3, 0.49-0.62μg/L; Quartile 4, >0.62μg/L (*vs*. quartile 1: *P <0.05, **P <0.01). (**D**) ROC curve analyses for the prediction of IR according to the betatrophin levels. (**E**) Circulating betatrophin levels in both IR and healthy women during EHC (*vs.* controls: ^∆^*P<*0.01, ^#^*P*<0.05; *vs.* 0 minutes: *P <0.01). (**F**) Circulating betatrophin levels in PCOS women with IR pre- and post-metformin treatment (*vs.* baseline: **P<0.01). (**G**) Circulating betatrophin levels during EHC pre- and post-metformin treatment (*vs*. baseline: ^∆^*P <* 0.01, ^#^*P* < 0.05; *vs.* 0 min: *P < 0.01).

Supplementary Table 1 summarizes the anthropometric and metabolic parameters of the subjects. As expected, homeostasis model assessment of insulin resistance (HOMA-IR), the area under the curve for glucose (AUC_glucose_) and insulin (AUC_insulin_) were significantly increased, while M-value was significantly decreased in PCOS women with IR compared with the controls. IR women had significantly elevated levels of fasting betatrophin as compared with the controls (0.57 ± 0.16 vs. 0.34 ± 0.16μg/L; *P* <0.01; Figure 1A) even after adjusting for body mass index (BMI). In the entire population, overweight/obese subjects (BMI ≥ 25 kg/m^2^) had significantly higher circulating betatrophin levels compared with lean individuals (BMI <25 kg/m^2^) (0.58 ± 0.17 *vs*. 0.43 ± 0.20μg/L; *P* <0.01; Figure 1B). Circulating betatrophin was correlated positively with BMI, age, waist-to-hip ratio (WHR), visceral fat percentage (Fat%), triglyceride (TG), total cholesterol (TC), low-density lipoprotein cholesterol (LDL-C), HbA1c, AUC_glucose_, AUC_insulin_, HOMA-IR, luteinizing Hormone (LH) and Free Androgen Index (FAI), but negatively with M-value (Supplementary Table 2). After controlling for other variables, betatrophin was still related to IR. Multivariate regression analyses demonstrated that LDL-C, LH and M-values were independently related factors influencing circulating betatrophin. In addition, the betatrophin levels showed a significant linear trend and were independently associated with IR especially when the concentrations were analyzed by row mean score differences and the Cochran-Armitage trend test (Supplementary Table 3). The odds ratios for M-value were significantly decreased along with increasing betatrophin quartiles (*P* for trend < 0.01; Figure 1C). Finally, the receiver operating characteristics (ROC) curve analysis revealed that the best cut off value for circulating betatrophin to predict IR was 0.44 μg/L (sensitivity 81.4 %, specificity 65.6 %, and AUC 0.762; Figure 1D).

### Effects of acute hyperinsulinemia on circulating betatrophin

EHCs were performed in all subjects. During the EHC, the steady-state (80-120 min) plasma glucose was clamped at 6 mmol. M-values were markedly lower in PCOS women than in controls (*P* < 0.01; Supplemental Table 1). In response to acute hyperinsulinemia, circulating betatrophin levels in the controls were significantly and rapidly dropped down from 0.37 ± 0.16 to 0.15 ± 0.09 μg/L at 80 min, then to 0.17 ± 0.08 μg/L at 100 min, and to 0.18 ± 0.16 μg/L at 110 min, and finally to 0.15 ± 0.09 μg/L (all *P* <0.01 *vs*. 0 min). In PCOS women with IR, circulating betatrophin concentrations were similarly dropped from 0.51 ± 0.12 to 0.19 ± 0.10 μg/L at 80 min, then to 0.17 ± 0.08 μg/L at 100 min, and to 0.19 ± 0.06 μg/L at 110 min, and finally to 0.19 ± 0.08 μg/L at 120 min (all *P*<0.01 *vs*. 0 min, Figure 1E).

### Effects of metformin treatment on circulating betatrophin in IR women

The data pre- and post-treatment with metformin were shown in Supplemental Table 4. In PCOS women with IR, after metformin administration, BMI, Fat%, TG, TC, HbA1c, fasting plasma insulin (FIns), 2-h plasma insulin after glucose overload (2-hIns), AUC_glucose_, AUC_insulin_, HOMA-IR, testosterone (TEST), FAI were declined significantly, whereas sex hormone- binding globulin (SHBG) increased (*P* <0.05 or *P*<0.01; Supplementary Table 4). During an OGTT, insulin levels at 0, 30, 60, and 120 min were lower than before metformin treatment (*P* <0.05 or *P*<0.01, Supplementary Figure 1A). In addition, M-values were significantly elevated (from 4.94 ± 1.90 to 5.74 ± 2.12 mg/kg/min at post-treatment 3 months, *P* < 0.01; and finally to 6.29 ± 2.03 mg/kg/min at post-treatment 6 months, *P* < 0.01; Supplementary Figure 1B). Importantly, after metformin treatment, circulating betatrophin levels in PCOS women were significantly decreased following increasing insulin sensitivity (from 0.54 ± 0.13 to 0.35 ± 0.12μg/L at post-treatment 3 months, and finally to 0.31 ± 0.10 μg/L at post-treatment 6 months, *vs.* baseline, both *P* <0.01; Figure 1F). After metformin treatments, the changes of betatrophin concentration patterns during EHC were similar with pretreatment (Figure 1G).

### Betatrophin expression in IR mouse tissues

**Figure 2:**
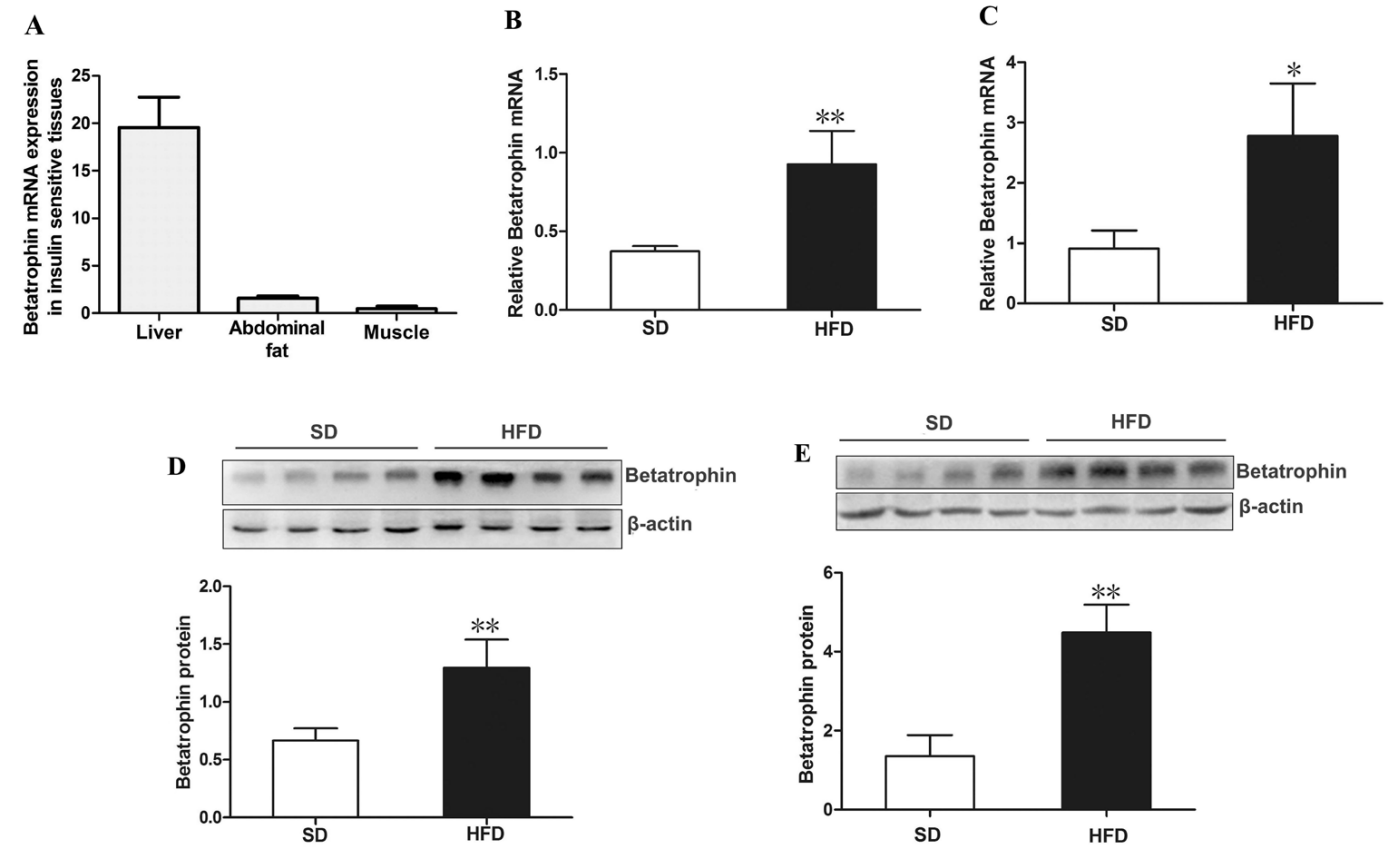
Expression of betatrophin at mRNA and protein levels in mice (**A**) Distribution of betatrophin mRNA expression in insulin target tissues from C57BL/6J mice (n=5). Betatrophin mRNA expression in liver (**B**) and fat (**C**) of SD- and HFD-fed mice. Betatrophin protein expression in liver (**D**) and fat (**E**) of SD- and HFD-fed mice (n = 5/group). SD, standard chow diet; HFD, high-fat diet; Data are presented as the mean ± SD. * *P* < 0.05, ** *P* < 0.01 *vs.* SD.

We further analyzed the expression patterns of betatrophin in insulin targeted tissues from C57BL/6J mice by using real-time quantitative PCR (RT-PCR). As shown in Figure 2A, betatrophin was expressed in liver, muscle and fat, while the highest expression levels were detected in liver and lowest levels in muscle. We found that high fat diet (HFD)-fed mice exhibited a significantly increased betatrophin expression at both mRNA and protein levels in liver (Figure 2B and 2D) and fat (Figure 2C and 2E) compared with standard chow diet (SD)-fed mice (*P* <0.05 or *P* <0.01).

### Effects of insulin, metformin and rosiglitazone on the expression of betatrophin in primary mouse hepatocytes (PHMs) and in the hepatocyte/ macrophage co-culture systems

**Figure 3:**
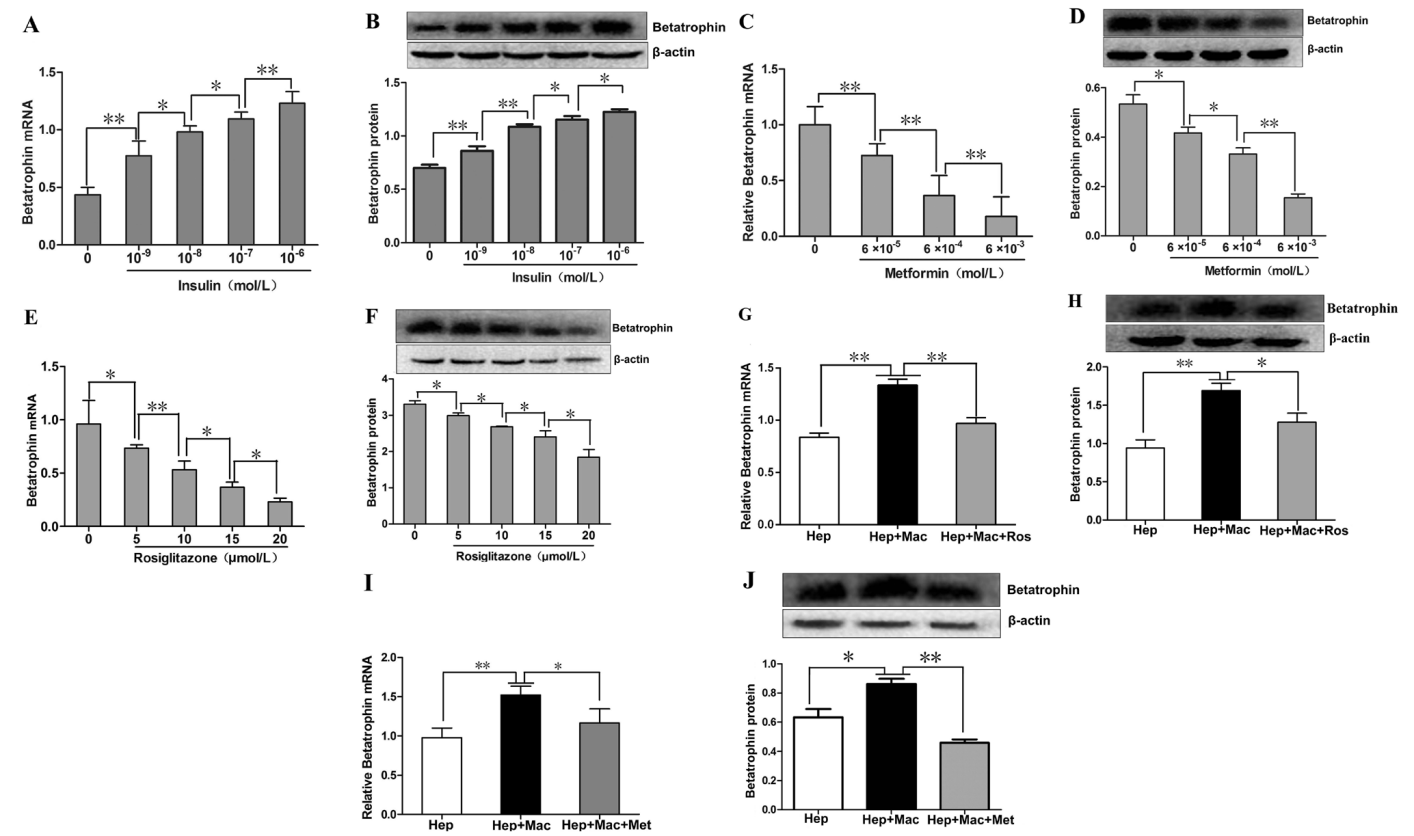
The effects of insulin, metformin and rosiglitazone on the expression of betatrophin in mouse hepatocytes and the hepatocyte/macrophage co-culture systems (**A**-**B**) Dose- dependent effects of insulin on betatrophin mRNA (A) and protein(B) expression in mouse hepatocytes. (**C**-**D**) Dose-dependent effects of metforminin the presence of 100 nM insulin on betatrophin mRNA (C) and protein (D)expression in mouse hepatocytes. (**E**-**F**) Dose- dependent effects of rosiglitazone in the presence of 100 nM insulin on betatrophin mRNA (E) and protein (F) expression in mouse hepatocytes. (**G**-**H**) Betatrophin mRNA (G) and protein (H) expression in cell lysates from the hepatocyte/macrophage co-culture systems with or without rosiglitazone treatment in the presence of 100 nM insulin. (**I**-**J**) Betatrophin mRNA (I) and protein (J) expression in cell lysates from the hepatocyte/macrophage co-culture systems with or without metformin treatment in the presence of 100 nM insulin. Hep, hepatocytes; Mac, macrophage; Ros, rosiglitazone. Data are presented as the means ± SE. * *P* < 0.05, **P < 0.01.

As shown in Figure 3A and 3B, we demonstrated that betatrophin expressions at mRNA and protein levels were increased dose-dependently by insulin stimulation in PHMs, but only a negligible amount of betatrophin protein was found in culture medium (data no shown). We then investigated whether metformin and rosiglitazone exerted a role on insulin-stimulated betatrophin expression in PHMs. Betatrophin mRNA and protein expressions in insulin-stimulated PHMs demonstrated a dose-dependent reduction after metformin (Figure 3C and 3D) and rosiglitazone (Figure 3E and 3F) treatment. In the co-culture systems of hepatocytes and macrophages, betatrophin expressions at mRNA and protein levels were significantly higher than that in hepatocyte culture alone (Figure 3G and H). However, the addition of rosiglitazone (Figure 3G and H) and metformin (Figure 3I and J) decreased the expression levels of betatrophin in the co-culture systems.

### Effects of betatrophin expression levels on insulin signaling *in vitro*

**Figure 4:**
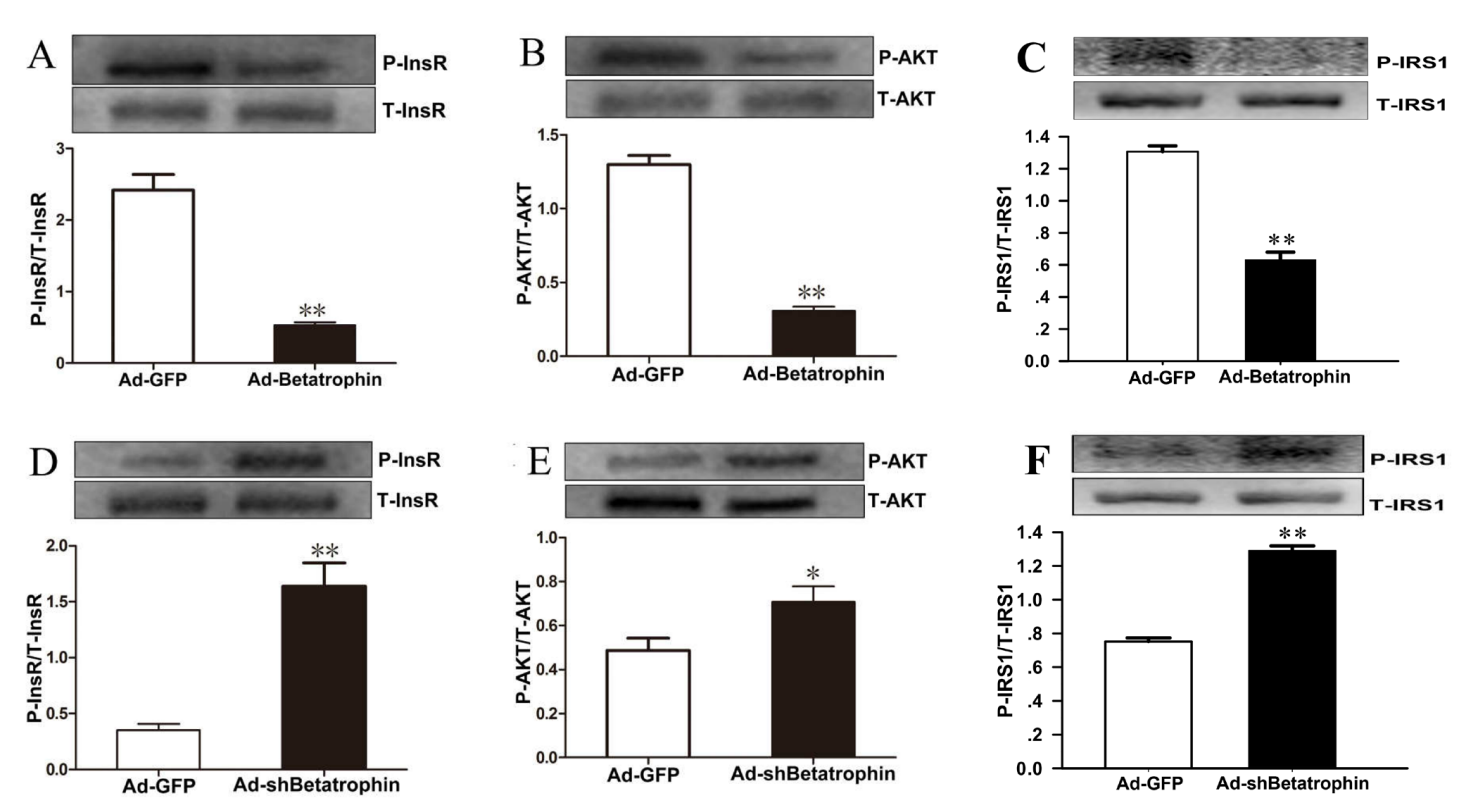
The effects of betatrophinexpression on insulin signaling in hepatocytes with insulin stimulation Primary hepatocytes were infected with or without recombinant adenovirus vectors encoding betatrophin (**A**-**C**) or *sh*RNA specific (**D**-**F**) or for betatrophin control green fluorescent protein (Ad-GFP) for 48 h, followed with 100 nmol/L insulin stimulation for 10 min. Western blot was conducted to examine the phosphorylation levels of InsR (A and D), AKT (B and E) and IRS-1 (C and F). Data are presented as the means ± SD. * *P* < 0.05, ** *P* < 0.01 *vs.* control.

To further explore a role of betatrophin in the regulation of insulin sensitivity, we investigated the effects of betatrophin over-expression and knockdown on phosphorylation of insulin receptor (InsR), protein kinase B (Akt) and insulin receptor substrate 1(IRS-1) in PHMs infected with recombinant adenoviruses used for overexpression of betatrophin (Ad-betatrophin) or kncodown of betatrophin (Ad-*sh*betatrophin) or vector that expresses a GFP (Ad-GFP). As expected, mRNA and protein expression was significantly increased in hepatocytes infected with Ad-betatrophin (Supplementaty Figure 2A and 2B) but reduced with Ad-*sh*betatrophin (Supplementary Figure 2C-2D). Importantly, we found that insulin-stimulated phosphorylation of InsR (Figure 4A), Akt (Figure 4B) and IRS-1 (Figure 4C) was significantly decreased in hepatocytes infected with Ad-betatrophin (all *P* < 0.01), whereas in hepatocytes infected with Ad-*sh*betatrophin, phosphorylation of InsR (Figure 4D), Akt (Figure 4E) and IRS-1 (Figure 4F) was significantly increased (*P* < 0.05 or *P* < 0.01). The phosphorylation of InsR and Akt without insulin-stimulation was shown in Supplementary Figure 3.

### Effects of human serum from IR women with metformin treatment on insulin signaling and betatrophin expression in hepatocytes

**Figure 5:**
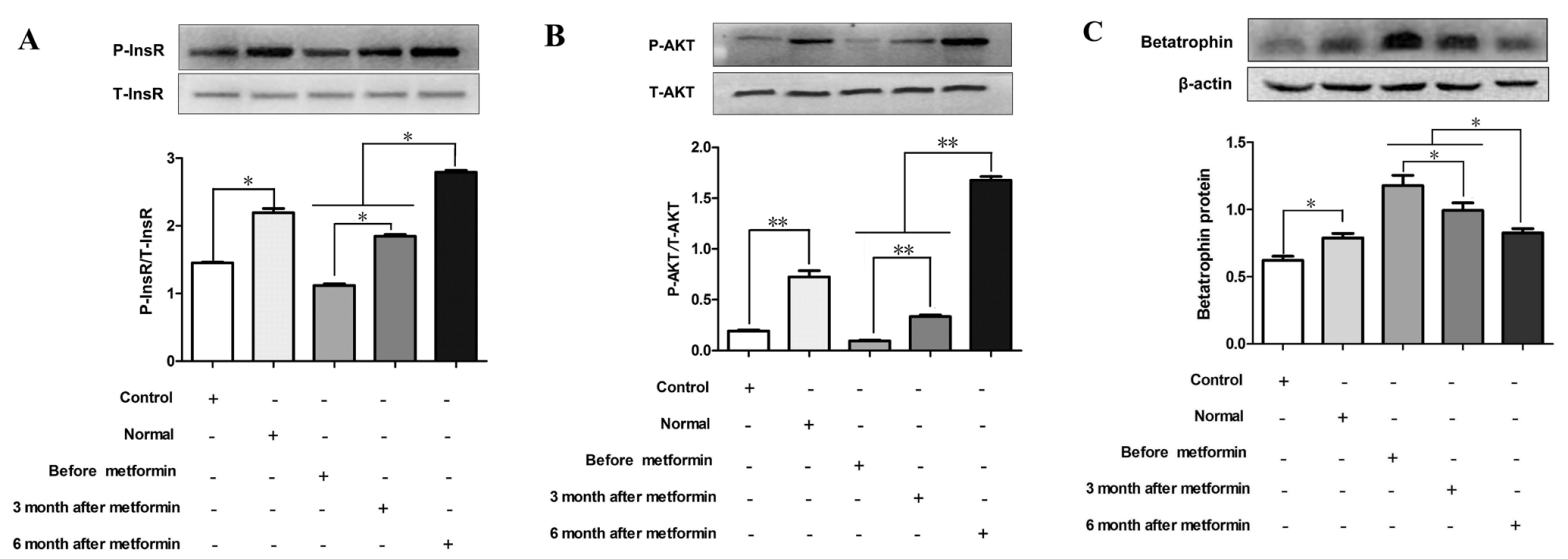
Effects of serum from normal and IR women on insulin signaling and betatrophin Mouse primary hepatocytes were treated by serum from normal subjects, IR women and IR women after 3 or 6 months of metformin treatment for 24hr. (**A**) Phosphorylation levels of insulin receptor (InsR). (**B**) Phosphorylation levels of Akt kinase (Akt). (**C**) Betatrophin protein expression. Each experiment was performed in three replicates. Control, saline; Normal, normal women serum; Data are shown as the means ± SD. * *P* <0.05, ** *P*<0.01.

As metformin has been shown to enhance insulin signaling [18], we examined the effects of serum from metformin-treated patients with both PCOS and IR on insulin signaling and betatrophin expression *in vitro*. In hepatocytes, the phosphorylation of InsR and Akt was significantly increased by sera from IR women after 6 months of metformin treatment compared with sera from these women before or after 3 months of treatment (*P* < 0.05 or *P* < 0.01, Figure 5A and 5B). Interestingly, sera from IR women treated with metformin for both 3 and 6 months led to a decrease of betatrophin protein expression in PHMs (*P* < 0.05, Figure 5C).

## DISCUSSION

In this study, we found that circulating betatrophin levels were significantly increased in PCOS women with IR compared with healthy women. Furthermore, we found that circulating betatrophin concentrations were correlated with IR markers in our cohort. Previous studies have reported the conflicting results concerning the association of betatrophin with IR [6-9]. The disparities may be due to the influence from confounding factors, including sample sizes, age, gender, glucose levels, and disease duration. Especially, it is unclear whether hypoglycemic agents will affect circulating betatrophin or not. To investigate the correlation between betatrophin and IR, confounding factors in the cohort of subjects should be limited as far as possible. In our cohort, PCOS subjects were young women with IR and were newly diagnosed and had not been treated with oral agents or diet control. Therefore, the effects of age, gender, disease duration and medicine were excluded.

Our findings are consistent with two recent reports [7, 8] and further confirm that betatrophin is associated with IR. We also observed that overweight/obese subjects had higher betatrophin levels than lean individuals, suggesting that betatrophin may be associated with obesity and metabolic disorders. Very recently, Calan et al. have reported that betatrophin levels were increased in PCOS women and were associated with HOMA-IR, high-sensitivity C-reactive protein (hs-CRP), and free-testosterone [19]. In addition, another study also found that circulating betatrophin was associated with IR in PCOS women [20]. Based upon the data from recent reports and our present study, it is reasonable to speculate that betatrophin may contribute to the development of chronic inflammation and IR.

We found a significant association between circulating betatrophin and triglyceride, which is similarity with the results of animal studies [21, 22] but contrary to the recent studies in T2DM [8, 10]. The inconsistent with our and that study may be due to the confounding factors, such as medical treatments. In the present study, we found that circulating betatrophin was correlated with TC and LDL-C. Quagliarini et al. have recently reported that a putative loss-of-function mutation in the betatrophin gene is associated with decreased LDL-C [23]. These data may implicate a novel role of betatrophin in lipid dysregulation.

Since PCOS women with androgen excess are at higher risk of IR compared with those with normal androgen levels [24, 25], it is important to analyze the relationship between betatrophin and androgen. It is well known that FAI is preferable to testosterone as a marker of androgen excess in PCOS women [25]. In the present study, we found that circulating betatrophin was positively correlated with FAI, suggesting that betatrophin may be associated with hyperandrogenism and play an important role in determining IR related to androgen in PCOS women with IR.

To better understand the relationship between betatrophin and IR, we performed EHC in women. The results showed that short-term hyperinsulinemia led to a rapid decrease of circulating betatrophin at the start of EHC. After that, betatrophin levels were constant at the steady-state of EHC. Importantly, there was a significant negative correlation between betatrophin and M-values in our cohort. As the gold standard for evaluating IR, This finding of EHC further confirms that betatrophin is related with IR and suggests an inhibitory effect of short-term hyperinsulinemia on betatrophin release.

Metformin has been shown to stimulate some cytokine release, such as glucagon-like peptide 1 [26]. To investigate the effect of metformin on betatrophin, we performed an interventional study in PCOS women with IR. We found that metformin treatment for 6 months resulted in a significant decrease of circulating betatrophin with a concomitant improvement in insulin sensitivity as shown by increasing M-values. However, this may not permit us to conclude that it was a direct effect of metformin on circulating betatrophin, or an indirect effect of decreasing BMI, increasing insulin sensitivity or decreasing androgen levels which were related to IR [25]. Therefore, further investigation is required to address this issue.

Betatrophin has been reported to be a liver-derived hormone. The concentration of betatrophin is higher than 250-fold in the liver compared with skeletal muscle and adipose tissues in humans [27]. Therefore, we investigated the cellular regulation of betatrophin expression and secretion by insulin or insulin sensitizer in hepatocytes and in the co-culture system of hepatocytes and macrophages. In PHMs, insulin increased dose-dependently betatrophin expression, but the protein accumulated intracellularly with only a tiny amount being release into culture media. Therefore, overexpression of betatrophin induced by insulin in PHMs unlikely contributed to extracellular/circulating concentrations of the protein as seen in *vivo* hyperinsulinemia. Recently, it has been reported that betatrophin secretion is depend on ANGPTL3 [23] and insulin downregulates ANGPTL3 mRNA and protein expression in hepatocytes [28]. In *vivo*, the insulin-induced decrease of circulating ANGPTL3 could result from down-regulation of gene expression in the liver [29]. Thereby, we hypothesize that the decrease of ANGPTL3 in hepatocytes by insulin may contribute to the decrease in medium or circulating betatrophin concentrations. To assess the direct effects of insulin sensitizer on betatrophin *in vitro*, we treated PHMs with metformin or rosiglitazone and observed a dose-dependent decrease in betatrophin expression. These results are consistent with the findings in interventional studies and suggest a transcriptional regulation of betatrophin in PHMs induced by insulin sensitizer. Although the regulation mechanism of betatrophin has not been clarified, it has been reported that adenosine 5‘-monophosphate (AMP)-activated protein kinase (AMPK) can modulate the expression of betatrophin in liver [30-31]. Therefore, we believe that metformin and rosiglitazone, two AMPK activators, can inhibit hepatic betatrophin expression in an AMPK-dependent manner.

Inflammatory cytokines secreted by macrophages have been demonstrated to augment tissue inflammation and to induce IR. To better understand the interaction between hepatocytes and macrophages on betatrophin expression, we co-cultured PHMs with macrophages. We found that the co-culture resulted in a significant increase of betatrophin expression, whereas the up-regulation of betatrophin was attenuated by the addition of rosiglitazone or metformin. The data showed a cell cross-talking between PHMs with macrophages and indicated that inflammatory cytokines secreted by macrophage had a specific role to increase betatrophin expression. Furthermore, we considered that the reduction of betatrophin expression in hepatocyte is likely due to the inhibitory effects of rosiglitazone or metformin on macrophage-secreted factors, because rosiglitazone and metformin have well-described anti-inflammatory effects [32, 33].

To directly observe the effects of betatrophin on insulin signaling, we over-expressed and knockdown betatrophin in PHMs and assessed the consequences on insulin action. Over-expression or knockdown of betatrophin impaired and improved insulin signaling by a reduction and an increase in insulin-stimulated InsR, Akt and IRS-1 phosphorylation in hepatocytes, respectively. The data are consistent with the *in vivo* findings that circulating betatrophin is positively correlated with HOMA-IR and provide the compelling evidence that betatrophin negatively regulates insulin sensitivity.

It has been reported that metformin treatment in PCOS women improves IR and corrects the associated endocrine and metabolic abnormalities [34]. Therefore, it is important to clarify the effects of metformin on insulin signaling and betatrophin expression *in vitro* combined with *in vivo*. We thus observed the effects of human serum from metformin-treated IR women on insulin signaling and betatrophin expression in PHMs. Our observation revealed that insulin-stimulated InsR and Akt phosphorylation in hepatocytes was significantly increased by serum from metformin-treated IR women, and the increase was accompanied by a decrease of betatrophin expression. Taking all these results together, we recognize that the increase of circulating betatrophin in IR subjects may have a potential role in the pathogenesis of IR.

Very recently, several studies have reported circulating betatrophin levels in PCOS women [35-37]. However, the results are inconsistent and PCOS women in those studies included both IR and no-IR subjects. In this study, only IR women were selected as study subjects and a series of interventional studies were performed in *vivo* and in *vitro*. Therefore, this study was adequately powered to demonstrate the association of betatrophin with IR in humans and used state-of-the-art methodology.

In conclusion, the present study provides the evidence indicating a significant increase of circulating betatrophin in untreated IR women and different effects of elevated insulin on betatrophin *in vivo* and *in vitro*. We also present novel data suggesting that metformin or rosiglitazone, possibly *via* a direct effect and/or an indirect effect of improved insulin sensitivity, decreases circulating levels and expression of betatrophin. Over-expression or knockdown of betatrophin impair or improve insulin signaling in hepatocytes, respectively. Furthermore, serum from metformin-treated IR women decreases betatrophin expression and reinforces insulin signal transduction in hepatocytes. Therefore, betatrophin is a useful marker of IR in humans.

## MATERIALS AND METHODS

### Cross-sectional studies

A total of 244 subjects including 100 healthy and 144 newly diagnosed PCOS women were recruited in this study. The diagnosis of PCOS was based on the 2003 Rotterdam consensus (The Rotterdam ESHRE/ASRM-sponsored PCOS consensus workshop group) [38]. 100 healthy women with regular periods and no hyperandrogenemia, hirsutism, or acne were recruited from the community or schools through advertisement, or routine medical check-up, and were used as the controls. Exclusion criteria included age > 35 years, BMI > 35 kg/m^2^, known cardiovascular disease, neoplasms, diabetes, hypertension, and renal impairment. The study was registered at ClinicalTrials.gov (ChiCTR-OCS-13003185). All subjects were Chinese Han population and given their written informed consent before entering the study.

### Interventional studies

A subset of 76 patients from the PCOS group received metformin treatment. Patients were selected for this study if they didn’t plan pregnancy, had no a history of current or recent (within 3 months) use of oral contraceptives, antidiabetics, or antiandrogens, and had no any contraindications to metformin therapy. To minimize gastrointestinal side effects, the dose of metformin was initiated from 500 mg once daily and increased to a maintenance dose of 2000 mg twice daily for 24 wk. Fasting blood samples were obtained at 0800 hour, and EHC was performed on day 0, on week 12 and on week 24 after metformin treatment.

### EHC studies and oral glucose tolerance test

EHC was performed in all subjects and in 76 PCOS women treated with metformin. In metformin-treated PCOS women, EHC was performed on day 0, on week 12 and 24 for three times as previously described [39, 40]. OGTT was performed in all subjects.

### Anthropometric, biochemical and betatrophin measurements

Waist circumference and hip circumference were measured for calculation of the WHR. The HOMA-IR was calculated using the following equations [41]: HOMA-IR = FIns (μU/mL) × fasting blood glucose (FBG) (mmol/L)/22.5. Insulin was measured by ELISA. Free fatty acid (FFA) was measured with a commercial kit. TC, high-density lipoprotein cholesterol (HDL-C), LDL-C, and TG were analyzed using an autoanalyzer. Serum sex hormone including LH, follicle- stimulating sormone (FSH), TEST and progestogen (Prog), prolactin (PRL) and estradiol (E_2_) were measured with electrochemi-luminescence immunoassay (Roche Diagnostics GmbH). Dehydroepiandrosteronesulfate (DHEA-S) and SHBG were performed using an automated analyzer (Abbott Laboratories, Abbott Park, IL). FAI was calculated as (testosterone/SHBG) × 100. Circulating betatrophin concentration was determined with an ELISA (Phoenix Pharmaceuticals Inc. Belmont, CA, USA) by using the manufacturer’s protocol.

### Generation of recombinant adenoviruses

The plasmid encoding betatrophin (PIRES2-EGFP-Betatrophin) was constructed as previously described [42]. The sequences were as follows: 5’-GGAAGATCTATGG CTGTGCTTGCTCTCTGCCTC-3’ (forward) and 5’-ACGCGTCGACTCAGGCTGG GAGGGCTGCTGTGT-3’ (reverse). The recombinant adenoviruses used for expression of betatrophin (Ad-betatrophin) and small hairpin RNA directed against the coding region of betatrophin (Ad-*sh*betatrophin) were generated using the AdEasy and the pAdxsi adenoviral vector system (SinoGenoMax Co. Ltd, Beijing, China) according to the instructions. The most effective sequence designed for knockdown of betatrophin is 5’-CAGCUCGAAGGUGUAAAGCTT-3’. A recombinant adenovirus vector that expresses a GFP (Ad-GFP) was used as a negative control [43].

### Animal preparation and treatment

Male C57BL/6J mice were purchased from Experimental Animal Center of Chongqing Medical University (Chongqing, China) at 7 weeks of age, acclimated for a week, and fed either a standard chow diet (13% fat) or a high fat diet (45% fat, Medicine Ltd., Jiangsu, China) for 12 weeks. Mice were anesthetized and sacrificed after fasted for 12 hours, and tissue samples were harvested and stored in liquid nitrogen until analysis. Animal procedures were approved by Chongqing Medical University Institutional Animal Care and Use Committee.

### Cell culture and co-culture

Hepatocytes were isolated from C57BL/6J mice as previously described [44]. PHMs were cultured in RPMI-1640 for 24 h. Cells were treated with insulin (0, 10^-9^, 10^-8^, 10^-7^, 10^-6^ mol/L), or rosiglitazone (0, 5, 10, 15, 20 µmol/L) or metformin (6×10^-5^, 6×10^-4^, 6×10^-3^ mol/L) plus insulin (100 nM) for 24 h, respectively. In another set of experiment, PHMs were treated with or without 1% of fasting serum from normal women (n=10) or metformin-treated PCOS women (pretreatment or post-treatment 3 or 6 months) (*n* =10) for 24 h. For the co-culture experiment, RAW 264.7 macrophages were seeded into upper layer of Trans-well insert plates and PHMs were seeded into the under layer of this plates for 40 h in 4ml fresh DMEM/F12 culture medium with or without 10 μM rosiglizone or 0.1 mM metformin. For vitro adenovirus infection, PHMs were grown in six-well plates and transfected with Ad-betatrophin, Ad-*sh*betatrophin or Ad-GFP for 48 h. The cell lysates were collected and stored at -80 °C.

### Real-time RT-PCR and western blot analysis

Real-time quantitative PCR was performed as described previously [45]. The primers used were: forward primer 5′-CACCTCT TATGGGCTCTCA-3′ and reverse primer 5′-AGTCTCTGCTGGATCTGTC-3′ for betatrophin; and forward primer 5′-AGAC CTCTATGCCAACACAGT-3′ and reverse primer 5′-TCGTACTCCTGCTTGCTGAT -3′ for β-actin. Protein analysis was performed with Western Blots as described previously [45]. Primary antibodies included anti-betatrophin (AbcamInc, UK); anti-AKT, anti-phostho-AKT, anti-insulin receptor, anti- phospho-InsR, anti-insulin receptor substrate 1, anti-phospho-IRS-1 (Cell Signaling, Beverly, MA, USA) and β-actin (Research Diagnostics Inc.).

### Statistical analysis

All analyses were performed with SPSS version 17.0. Data were expressed as means ± SD or SE, or median (interquartile range). Comparisons between groups were performed by repeated measures ANOVA, unpaired *t* test, or paired *t* test. Pearson correlation analysis was used to evaluate the relationship of betatrophin with other covariates. Multiple linear regression analyses using a stepwise method (probability for entry ≤ 0.05) for the introduction of independent variables were used to identify the main determinants of betatrophin levels and among the variables showing a statistically significant correlation in univariate analysis. The association of betatrophin with M-value was examined by binary logistic regression analysis. The distribution of betatrophin in the pooled data was further divided into tertiles, and the significant trends across increasing tertiles were estimated by row mean scores and the Cochran-Armitage trend test. ROC curves of betatrophin levels were constructed to determine the optimal cutoff point for the diagnosis of IR. *P* values < 0.05 were considered as significant.

## SUPPLEMENTARY MATERIALS FIGURES AND TABLES





## Abbreviations

PCOS: polycystic ovary syndrome women
IR: insulin resistance
EHC: euglycemic–hyperinsulinemic clamp
RT-PCR: real-time quantitative PCR
BMI: body mass index
WHR: waist-to-hip ratio
Fat%: visceral fat percentage
LDL-C: low- density lipoprotein cholesterol
AUC_glucose_: the area under the curve for glucose
AUC _insulin_: the area under the curve for insulin
FAI: Free Androgen Index
HOMA-IR: homeostasis model assessment of insulin resistance
