# Supplementary material for: Circulating betatrophin is associated with insulin resistance in humans: cross-sectional and interventional studies in vivo and in vitro
Source: Oncotarget. 2017 Oct 16;8(57):96604–14. doi: 10.18632/oncotarget.21852 (PMC5722507; doi:10.18632/oncotarget.21852)
Supplement: Supplementary file 1 [file oncotarget-08-96604-s001.pdf]

Circulating betatrophin is associated with insulin resistance in humans: cross-sectional and interventional studies *in vivo* and *in vitro*

SUPPLEMENTARY MATERIALS

For Supplementary Tables see in Supplementary Files.

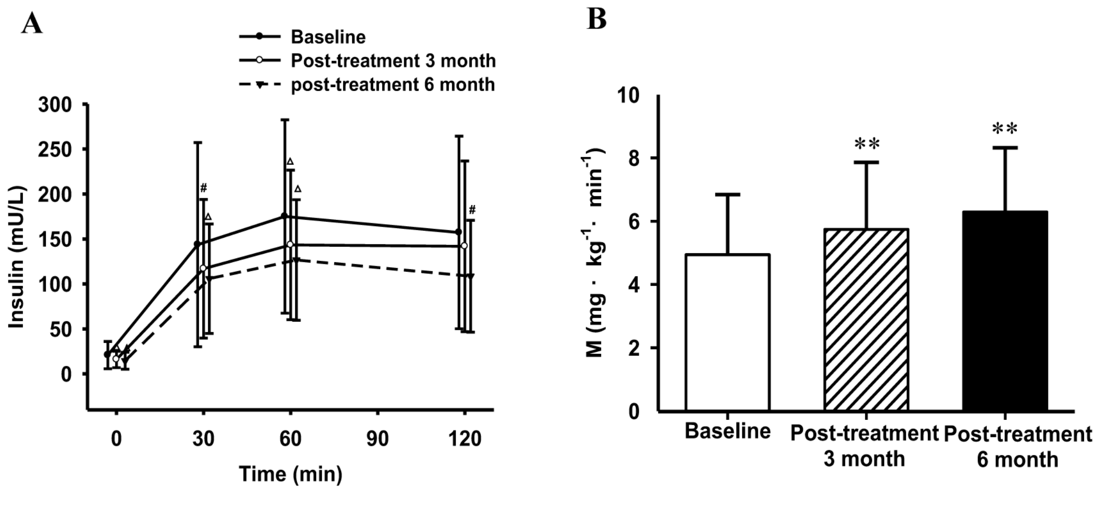

Supplementary Figure 1: Effects of metformin treatment on insulin sensitivity in PCOS women. (A) Plasma insulin levels in PCOS women during an OGTT pre- and post-metformin treatment (*vs.* baseline: $^{\Delta}P<0.01$, $^{\#}P<0.05$). (B) M values in PCOS women during EHC pre- and post-metformin treatment (*vs.* baseline: $^{**}P<0.01$).

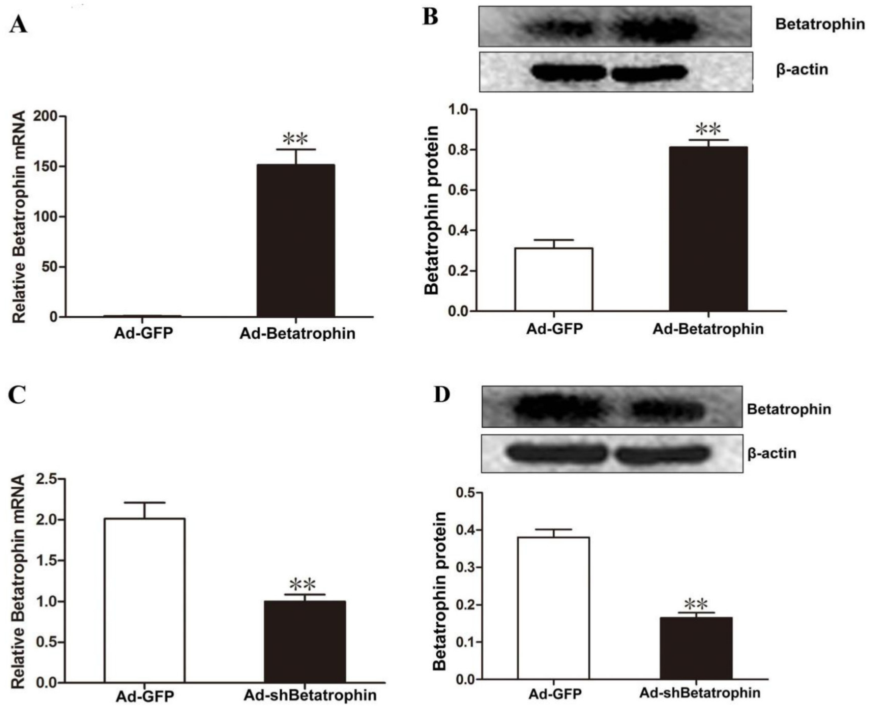

Supplementary Figure 2: Betatrophin expression in mouse hepatocytes. (A and B) The mouse primary hepatocytes were infected with recombinant adenovirus vectors encoding betatrophin for 48 h. Quantitative RT-PCR and western blotting were conducted to examine mRNA (A) and protein (B) expression of betatrophin. (C and D) The mouse primary hepatocytes were infected with recombinant adenovirus vectors encoding *sh*RNA specific for betatrophin for 48 h. Quantitative RT-PCR and western blotting were conducted to examine mRNA (C) and protein (D) expression of betatrophin. Data are presented as the mean ± SD. *vs.* Ad-GFP, ** $P<0.01$.

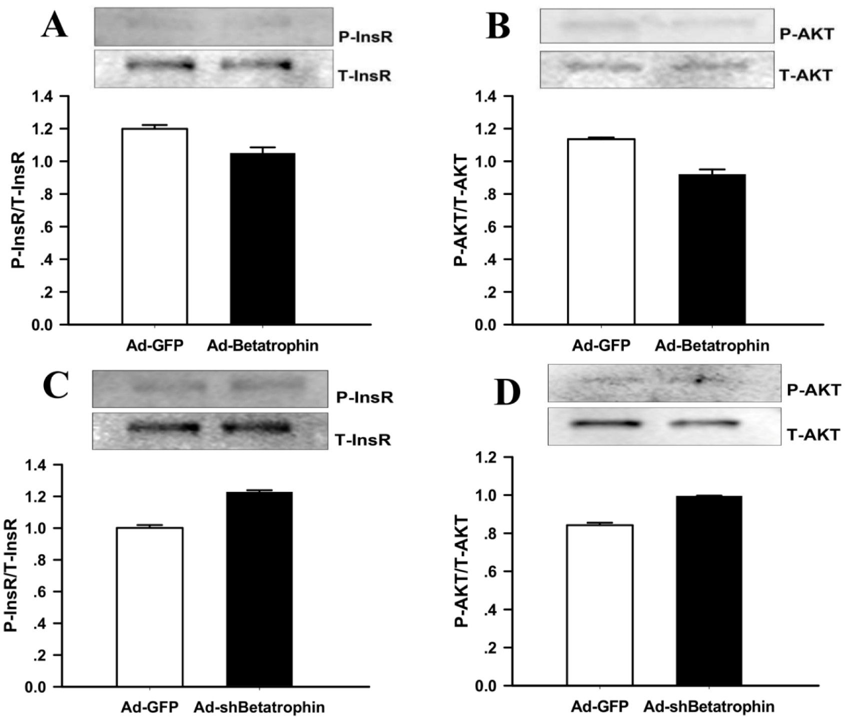

Supplementary Figure 3: The effects of betatrophin expression level on insulin signaling on basal status. Primary hepatocytes were infected with or without recombinant adenovirus vectors encoding betatrophin (A and B) or *sh*RNA specific (C and D) or for betatrophin control green fluorescent protein (Ad-GFP) for 48 h. Western blot was conducted to examine the phosphorylation levels of InsR (A and C) and AKT (B and D). Data shows no significant.
