# Supplementary material for: Circulating betatrophin is associated with insulin resistance in humans: cross-sectional and interventional studies in vivo and in vitro
Source: Oncotarget. 2017 Oct 16;8(57):96604–14. doi: 10.18632/oncotarget.21852 (PMC5722507; doi:10.18632/oncotarget.21852)
Supplement: Supplementary file 2 [file oncotarget-08-96604-s002.docx]

**Supplementary Table 1:** Clinical and metabolic parameters of PCOS women and control subjects

| Variable | Controls  (n=100) | PCOS  (n=144) |
| --- | --- | --- |
|  |  |  |
| Age (yr) | 25.7 ± 2.3 | 26.5 ± 4.9 |
| Body weight (kg) | 51.3 ± 7.3 | 62.3 ± 12.0** |
| BMI (kg/m^2^) | 20.5 ± 2.6 | 25.2 ± 4.6** |
| WHR | 0.79 ± 0.05 | 0.89 ± 0.29** |
| Fat (%) | 26.70 ± 5.49 | 36.50 ± 8.93** |
| TG ( mmol/L) | 0.79 (0.57-1.28) | 1.32 (0.98-1.97)** |
| TC (mmol/L ) | 3.82 ± 0.99 | 4.53 ± 0.94** |
| HDL-C (mmol/L) | 1.20 ± 0.33 | 1.43 ± 1.38* |
| LDL-C (mmol/L) | 2.15 ± 0.86 | 2.61 ± 0.82** |
| FFA (umol/L) | 0.56 ± 0.27 | 0.60 ± 0.34 |
| FBG (mmol/L) | 4.45 ± 0.46 | 5.12 ± 1.14** |
| 30 min-OGTT | 7.36 ± 1.55 | 9.12 ± 2.10** |
| 60 min-OGTT | 5.96 ± 1.49 | 8.83 ± 2.85** |
| 2h-OGTT (mmol/L) | 5.38 ± 1.08 | 7.81 ± 2.89** |
| FIns (mU/L) | 7.02 (6.10-8.62) | 16.26 (9.49-23.99)** |
| 2h-Ins (mU/L) | 36.55 (22.39-58.17) | 123.70 (73.45-170.00)** |
| HbA1c (%) | 5.17 ± 0.25 | 5.45 ± 0.53** |
| AUC_glucose_ | 11.98 ± 2.20 | 16.66 ± 4.67** |
| AUC_insulin_ | 124.9 ± 99.2 | 255.1 ± 167.9** |
| M-value (mg/kg/min) | 10.16 ± 2.54 | 5.56 ± 2.57** |
| HOMA-IR | 1.54 ± 0.65 | 4.43 ± 3.66** |
| Betatrophin (μg/L) | 0.34 ± 0.16 | 0.57 ± 0.16** |
| DHEA-S (μg/dl) | 194.1 ± 85.7 | 211.6 ± 94.4 |
| PRL (mIU/L) | 324.8 ± 97.9 | 356.4 ± 167.4* |
| E2 (pmol/L) | 183.5 (117.4-260.8) | 194.1 (116.3-266.6) |
| LH (IU/L) | 4.76 ± 2.60 | 9.29 ± 5.95** |
| FSH (IU/l) | 8.02 ± 1.91 | 7.40 ± 2.15 |
| Prog (nmol/L) | 2.7 ± 1.1 | 2.6 ± 1.6 |
| SHBG (nmol/L) | 61.2 ± 24.7 | 40.2 ± 25.7** |
| TEST (nmol/L ) | 1.82 ± 0.74 | 2.65 ± 1.46** |
| FAI | 2.59 (1.81-4.84) | 7.05 (4.26-11.43)** |

PCOS, polycystic ovary syndrome; BMI, body mass index; WHR, waist-to-hip ratio; Fat %, visceral fat percentage; TG, triglyeride; TC, total cholesterol; HDL-C, high-density lipoprotein cholesterol; LDL-C, low-density lipoprotein cholesterol; FFA, free fatty acids; FBG, fasting blood glucose; 30 min-OGTT, 30-min post-glucose load blood glucose; 60 min-OGTT, 60-min post-glucose load blood glucose; 2h-OGTT, 2-h post-glucose load blood glucose; FIns, fasting plasma insulin; 2-hIns, 2-h plasma insulin after glucose overload; AUC_glucose_, the area under the curve for glucose; AUC_insulin_, the area under the curve forinsulin; HOMA-IR, HOMA-insulin resistance index; DHEA-S: Dehydroepiandrosterone sulfate; PRL, Prolactin; E2, estradiol; LH, Luteinizing hormone; FSH, Follicle-stimulating hormone; Prog, Progesterone; SHBG, Sex-hormone binding globulin; TEST, testosterone; FAI, free androgen index = T(nmol/l) / SHBG (nmol/l) × 100. Data are mean ± SD or median. **P* <0.05, ***P* < 0.01 compared with controls.

|  | Simple | |  | Adjusted BMI | |  | Multivariate | | |
| --- | --- | --- | --- | --- | --- | --- | --- | --- | --- |
|  | *r* | *P* |  | *r* | *P* |  | b | *P* | |
| BMI | 0.322 | <0.001 |  | ------- | ------- |  | ------- | | ------- |
| Age | 0.148 | 0.021 |  | 0.147 | 0.024 |  | ------- | | ------- |
| WHR | 0.163 | 0.011 |  | 0.110 | 0.091 |  | ------- | | ------- |
| Fat (%) | 0.327 | <0.001 |  | 0.083 | 0.202 |  | ------- | | ------- |
| TG | 0.155 | 0.015 |  | 0.118 | 0.069 |  | ------- | | ------- |
| TC | 0.291 | <0.001 |  | 0.242 | <0.001 |  | ------- | | ------- |
| HDL-C | 0.037 | 0.564 |  | 0.023 | 0.721 |  | ------- | | ------- |
| LDL-C | 0.270 | <0.001 |  | 0.261 | <0.001 |  | 0.053 | | <0.001 |
| FFA | 0.027 | 0.670 |  | 0.011 | 0.862 |  | ------- | | ------- |
| HbA1c | 0.139 | 0.030 |  | 0.057 | 0.380 |  | ------- | | ------- |
| AUC_glucose_ | 0.270 | <0.001 |  | 0.159 | 0.014 |  | ------- | | ------- |
| AUC_insulin_ | 0.292 | <0.001 |  | 0.176 | 0.007 |  | ------- | | ------- |
| HOMA-IR | 0.291 | <0.001 |  | 0.148 | 0.023 |  | ------- | | ------- |
| M-value | -0.375 | <0.001 |  | -0.222 | 0.001 |  | -0.014 | | <0.001 |
| DHEA-S | 0.115 | 0.073 |  | 0.073 | 0.265 |  | ------- | | ------- |
| E2 | 0.102 | 0.118 |  | 0.067 | 0.307 |  | ------- | | ------- |
| LH | 0.277 | <0.001 |  | 0.281 | <0.001 |  | 0.008 | | <0.001 |
| FSH | -0. 034 | 0.601 |  | 0.043 | 0.507 |  | ------- | | ------- |
| PRL | 0.052 | 0.415 |  | 0.014 | 0.828 |  | ------- | | ------- |
| Prog | -0.035 | 0.589 |  | -0.034 | 0.605 |  | ------- | | ------- |
| FAI | 0.307 | <0.001 |  | 0.203 | 0.002 |  | ------- | | ------- |

**Supplementary Table 2:** Linear regression analysis of variables associated with circulating betatrophin levels

In the multiple linear stepwise regression analysis, the values included for analysis were WHR, TG, LDL-C, M-value, LH, FAI.

**Supplementary Table 3:** Row Mean Scores and Cochran–Armitage Trend Test of the impact of circulating betatrophin levels on insulin resistance

| Model adjusted | PCOS | |
| --- | --- | --- |
|  | 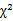 | *P*-value |
| Row Mean Scores Test | 31.2609 | <0.001 |
| Cochran-Armitage Trend Test | -5.2526 | <0.001 |

The circulating betatrophin levels of all subjects were cut-off, and adjusted for age, BMI, WHR, lipid profile, DHEA-S, E2, LH, FSH, PRL, Prog and FAI.

**Supplementary Table 4:** Clinical, hormonal, and metabolic features of women with PCOS before and after metformin treatment

| Variable | baseline | Post-treatment  3 months | Post-treatment  6 months |
| --- | --- | --- | --- |
| BMI (kg/m^2^) | 25.97 ± 4.25 | 24.83 ± 4.12** | 24.01 ± 3.85** |
| WHR | 0.88 ± 0.09 | 0.87 ± 0.06 | 0.86 ± 0.06 |
| Fat (%) | 37.75 ± 7.49 | 35.79 ± 7.22** | 33.79 ± 7.21** |
| TG ( mmol/L) | 1.81 ± 1.33 | 1.56 ± 1.22 | 1.47 ± 1.34* |
| TC ( mmol/L ) | 4.59 ± 0.86 | 4.34 ± 0.82 | 4.33 ± 0.80 |
| HDL-C (mmol/L) | 1.39 ± 0.28 | 1.23 ± 0.30 | 1.23 ± 0.26 |
| LDL-C (mmol/L) | 2.67 ± 0.85 | 2.50 ± 0.75 | 2.48 ± 0.80 |
| FFA (umol/L) | 0.64 ± 0.44 | 0.52 ± 0.19 | 0.60 ± 0.34 |
| HbA1c (%) | 5.56 ± 0.64 | 5.36 ± 0.53** | 5.14 ± 0.62** |
| FBG (mmol/L) | 5.28 ± 1.46 | 5.05 ± 0.86 | 5.00 ± 0.83 |
| 2h-OGTT (mmol/L) | 8.28 ± 3.33 | 7.91 ± 2.01 | 7.55 ± 2.15 |
| FIns (mU/L) | 20.82 ± 15.15 | 16.02 ± 9.15** | 14.56 ± 9.39** |
| 2h-Ins (mU/L) | 157.2 ± 107.1 | 141.8 ± 94.9 | 108.5 ± 62.2** |
| AUC_glucose_ | 17.80 ± 5.41 | 17.07 ± 3.33 | 16.38 ± 3.56* |
| AUC _insulin_ | 258.6 ± 162.0 | 242.1 ± 134.8 | 208.1 ± 103.7** |
| HOMA-IR | 4.07(2.64-6.16) | 3.02(1.94-4.40)** | 2.82(1.72-4.04)** |
| Betatrophin (μg/l) | 0.54 ± 0.13 | 0.35 ± 0.12** | 0.31 ± 0.10** |
| M value | 4.9 ± 1.90 | 5.74 ± 2.12** | 6.29 ± 2.03** |
| SHBG (nmol/L) | 36.31 ± 23.17 | 49.59 ± 36.30 | 59.47 ± 40.76* |
| TEST ( nmol/L ) | 2.35 ± 1.02 | 2.20 ± 1.06 | 2.02 ± 1.05* |
| DHEA-S (μg/dl) | 220.8 ± 83.09 | 251.80 ± 124.36 | 233.43 ± 99.46 |
| E2(pmol/l) | 195.2 ± 114.5 | 223.0 ± 195.4 | 230.4 ± 182.1 |
| LH (IU/l) | 8.07 ± 4.98 | 7.94 ± 6.82 | 6.84 ± 4.35 |
| FSH (IU/l) | 7.19 ± 1.97 | 6.91 ± 2.17 | 7.05 ± 2.28 |
| PRL (mIU/L) | 348.1 ± 170.2 | 390.9 ± 211.5 | 399.6 ± 218.9 |
| Prog (nmol/L) | 2.44 ± 1.56 | 2.35 ± 1.29 | 2.52 ± 1.26 |
| FAI | 8.89 ± 7.38 | 7.07 ± 6.41 | 5.10 ± 5.30** |

BMI, body mass index; WHR, waist-to-hip ratio; Fat%, visceral fat percentage; TG, triglyeride; TC, total cholesterol; HDL-C, high-density lipoprotein cholesterol; LDL-C, low-density lipoprotein cholesterol; FFA, free fatty acid; FBG, fasting blood glucose; 2h-BG, 2 h post-glucose load blood glucose; FIns, fasting plasma insulin; 2h-Ins, 2-h plasma insulin after glucose overload; AUC_glucose_, the area under the curve for glucose; AUC_insulin_, the area under the curve for insulin; HOMA-IR, HOMA- nsulin resistance index; M, whole body glucose uptake rate; SHBG, sex hormone- inding globulin; TEST, testosterone; DHEA-S, dehydroepiandrosterone- ulfate; LH, luteinizing hormone; FSH, follicular stimulating hormone; Prog, Progesterone. Free androgen index (FAI) = T (nmol/l)/SHBG (nmol/l)×100. Values were given as means ± SD or median (interquartile range). **P* <0.05, ***P* <0.01 compared with baseline.
